# The Modeling of a Single-Electron Bipolar Avalanche Transistor in 150 nm CMOS

**DOI:** 10.3390/s25113354

**Published:** 2025-05-26

**Authors:** Abderrezak Boughedda, Lucio Pancheri, Luca Parmesan, Leonardo Gasparini, Gabriele Quarta, Daniele Perenzoni, Matteo Perenzoni

**Affiliations:** 1 Fondazione Bruno Kessler, 38123 Trento, Italy; luparmesan@fbk.eu (L.P.); gasparini@fbk.eu (L.G.); 2 Industrial Engineering Department, University of Trento, 38123 Trento, Italy; lucio.pancheri@unitn.it; 3 Sony Europe Technology Development Centre, 38123 Trento, Italy; gabriele.quarta@sony.com (G.Q.); daniele.perenzoni@sony.com (D.P.); matteo.perenzoni@sony.com (M.P.)

**Keywords:** avalanche, SEBAT, TCAD, THz FET detector

## Abstract

This paper addresses the complex behavior of Single-Electron Bipolar Avalanche Transistors (SEBATs) through a comprehensive modeling approach. TCAD simulations were used to analyze the behavior of the device during avalanche pulses triggered by electron injection. The simulations consider the avalanche process and charge flow and include the parasitic capacitances and resistances. A SPICE model is proposed using parameters extracted from the TCAD simulations. Both TCAD and SPICE simulations are validated against experimental results obtained on 150 nm CMOS devices and are employed to provide a clear understanding of the phenomena observed experimentally during SEBAT operation. The impact of parasitic elements on device operation is studied using simulations. This work enables the optimization of SEBAT devices and their integration in circuits for better signal-to-noise ratios, efficiency, and potential applications in sensing and digitizing low-level signals.

## 1. Introduction

The observation of charge quantization in electronic devices has long been a topic of interest, particularly in the context of noise and signal processing [1]. In traditional bipolar junction transistors (BJTs) [2], charge quantization effects are often masked by other noise sources and can only be indirectly inferred through phenomena like shot noise [3]. However, recent advancements in device design have led to the development of novel transistor configurations that can directly exploit these quantization effects like Single-Electron Transistors (SETs) [4,5,6].

One such innovation is the Single-Electron Bipolar Avalanche Transistor (SEBAT), which operates at room temperature and can be fabricated using standard CMOS technology [7]. The SEBAT features a base-collector junction, which has a structure resembling an avalanche photodiode [8], and an injector region, which forms a junction with the base and is used to inject electrons towards the avalanche junction. The base-collector junction operates in Geiger mode for high-sensitivity detection of individual electrons reaching the high-field region. This mechanism enables the direct conversion of weak input signals into countable digital outputs, eliminating the need for analog amplification stages while minimizing the overall complexity [9]. SEBATs have been proposed for various applications, from the readout of weak currents to the generation of random numbers [10].

Among the proposed applications, SEBATs have shown promise as a means to fabricate a compact integrated analog-to-digital converter for the readout of a THz FET detector. In this context, the proposed device produces Geiger-mode avalanche pulses at a frequency varying with the power of the THz radiation coupled to the antennas. Counting the rate of avalanche pulses enables the conversion of the captured THz power to a digital signal. This approach has proven effective for THz detection. However, the electron-to-pulse conversion efficiency achieved in the experiment is poor, with the rate of avalanche pulses a factor of 10^−4^ lower than the rate of injected electrons [11]. This inefficiency contrasts starkly with the 70% efficiency reported in reference [7]. A very low injector efficiency of the order of 2·10^−4^% was also observed in [12]. Although in [12], this inefficiency is attributed to electron recombination in the transistor base; such a large inefficiency can hardly be explained by recombination in the thin base region. On the other hand, an accurate analysis of SEBAT transient operation in the Geiger mode has not been conducted so far.

This article presents a modeling approach to improve the current understanding of SEBAT operation and provides indications to enhance its performance. Section 2 presents a selection of experimental results obtained on a test structure formed by a SEBAT coupled with a THz detector. In Section 3, technology computer-aided design (TCAD) simulations analyze the transient behavior of the device during electron injection pulses. The simulations consider the avalanche process, charge flow, and the effect of various parasitic elements such as capacitances and resistances. A SPICE model, using parameters extracted from TCAD simulations, is proposed in Section 4 to reproduce the main physical phenomena observed in the SEBAT in a lightweight model suitable for circuit simulations. Both the TCAD simulations and the SPICE model are validated against experimental results, and the main findings are discussed in Section 5.

## 2. Test Devices and Experimental Results

**Figure 1:**
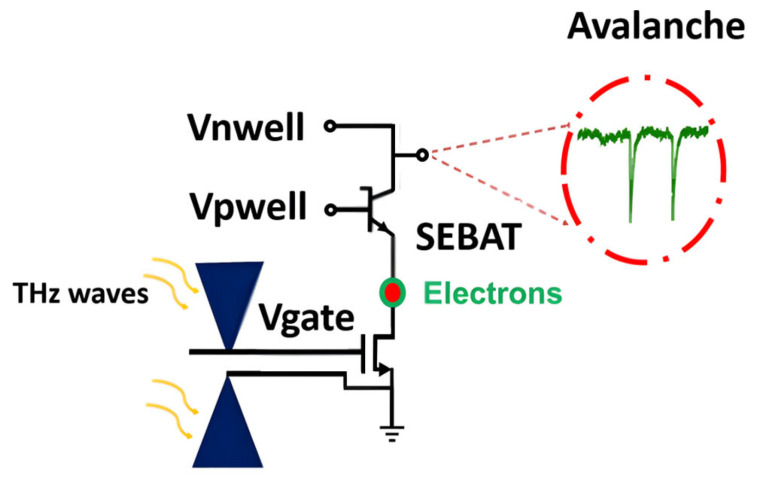
Simplified schematic of a Single-Electron Transistor coupled to a THz detector according to the power-to-frequency converter principle.

The characteristics of a CMOS-integrated SEBAT were analyzed experimentally using a test device designed for THz radiation detection. The proposed device, called THz Single-Electron Transistor (THzSET), consists of three main components: an NMOS transistor integrated with a bow-tie antenna as the THz detector; a SEBAT that amplifies the detector’s small signal, generating a stream of current pulses; and a readout circuit that enables the counting of avalanche pulses. The schematic of the proposed device is shown in Figure 1. The pixel was implemented in a commercial 150 nm CMOS process. More details about the detector design can be found in [9]. Although specifically designed for THz radiation sensing, all the voltages can be externally controlled, making this device suitable as a test bench for the characterization of the main device’s characteristics.

**Figure 2:**
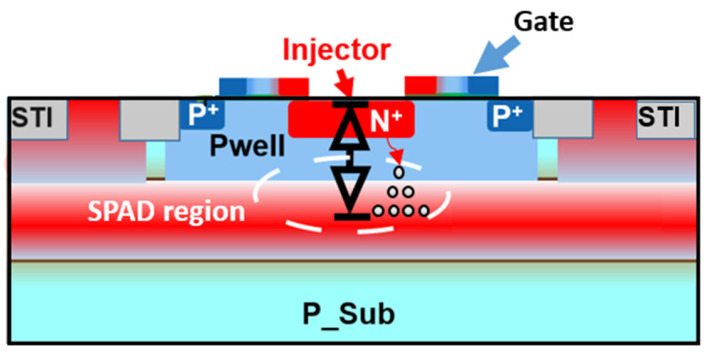
Cross-section of the implemented SEBAT in 150 nm CMOS.

The SEBAT structure resembles a single-photon avalanche diode (SPAD) [13], but it includes an extra injector region, transforming it into a bipolar junction transistor (BJT) configuration, as illustrated in Figure 2. The dimensions of the device are as follows: the emitter (n+) area is ~2 µm^2^; the base (pwell) has an octagonal shape with an area of ~18 µm^2^; and the collector (deep-nwell) area is 400 µm^2^. To operate in Geiger mode, a specific biasing scheme is employed: Nwell (collector) is biased at a high positive voltage (typically around 22 V), while Pwell (base) is connected to ground. The n+/Pwell (emitter-base) junction is forward biased by applying a negative voltage to the emitter, which injects electrons into the base towards the high-field avalanche region. This biasing configuration creates a unique detection mechanism where a single electron reaching the high-field region can trigger an avalanche multiplication process. When an electron enters the base-collector junction, it initiates an avalanche breakdown event, generating a large, detectable current pulse.

The gate on top of the SEBAT primarily serves as a mask for N+ injector implantation. The gate may have some minor parasitic effects on the SEBAT operation by slightly increasing the injector-base capacitance.

The SEBAT was initially characterized in linear mode and subsequently in Geiger mode operation. The characterization curves were obtained by biasing the MOSFET gate at 1.8 V to ensure it was fully on. This was necessary due to the series connection between the THz detection MOSFET and the SEBAT and due to the fact that the SEBAT injector can only be biased through the MOSFET. An equivalent resistance of 2.7 kΩ was estimated for the MOSFET at this gate bias voltage. The collector current was measured as a function of the base-emitter voltage, with the collector biased at 5 V, to avoid impact ionization from affecting the measurement. Then, an external series resistor was applied to the collector, and the collector-base junction was biased in the Geiger mode at 2.5 V excess bias. The stream of Geiger pulses was acquired using an oscilloscope, and the device was kept in the dark. The pulse rate as a function of base-emitter voltage was recorded for different voltages applied to the detector gate.

**Figure 3:**
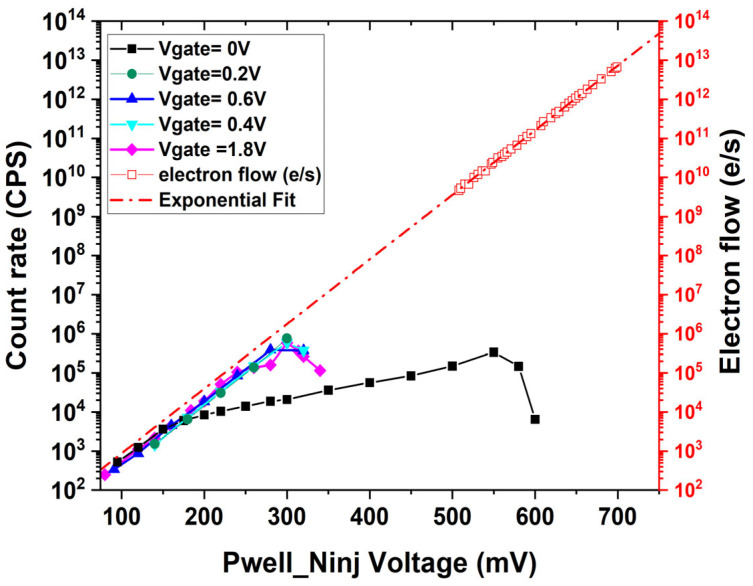
Count rate measured on the SEBAT in the Geiger mode and the electron flow rate estimated from the emitter current in linear mode.

Figure 3 shows the electron flow rate of the emitter in linear operation, estimated from the I-V curves, and the output pulse rate was measured for the SEBAT as a function of the applied base-emitter voltage. The alpha factor (α), which is estimated from common-emitter current gain β, is used to calculate emitter current I_c_ from collection current I_e_ (I_c_ = αI_e_), where α is very close to 1 in this device. This plot provides a direct comparison of the electron injection rate to the avalanche event detection rate for the same base-emitter voltage. The count rate shows saturation at approximately 1 MHz due to the recharge time, which is limited by the adoption of an external quenching resistor. The discharge and recharge process requires approximately 1 μs, which is consistent with the observed saturation at ~1 MHz. The avalanche discharge process itself occurs in ~1 nanosecond and is not affected by the quenching resistor value. The 10 MΩ quenching resistor was chosen as a compromise between ensuring complete avalanche quenching and allowing counts up to the MHz range while maintaining negligible afterpulsing. A smaller resistor would reduce the recharge time but could increase the probability of afterpulsing, especially given the relatively large capacitance in our external quenching configuration. This optimization is particularly important for THz applications, where jitter has a minimal impact due to the longer measurement time scales, while maintaining low afterpulsing and ensuring signal levels significantly exceed the dark count rate are critical for measurement accuracy.

For gate voltages above 0.2 V, the count rate exhibits an exponential increase with the applied Pwell (base) voltage, proportional to the diode’s current–voltage characteristics. In contrast, when applying a gate voltage of 0 V to the gate of the FET, the count rate increases with a lower slope, thus deviating from the characteristics measured in linear operation.

**Figure 4:**
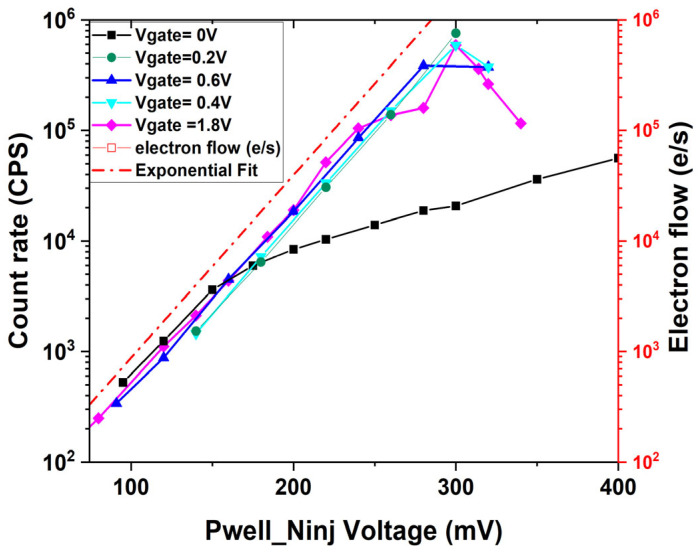
Zoomed graph of the count rate measured on the SEBAT in the Geiger mode and the electron flow rate estimated from the emitter current.

The analysis utilized regression on experimental data points in the linear region where the PN junction exhibits an ideality factor close to 1, corresponding to ideal diode behavior. This regression approach was employed to estimate values at lower voltage ranges where direct measurements are not reliable due to setup limitations and external interference. The Electron Detection Efficiency (EDE) can be defined as the ratio between the electron flow rate and the count rate at the same bias voltage. By comparing the extrapolation of the electron flow rate to low base-emitter voltages with the count rate in the Geiger mode, an electron detection efficiency of around 26% can be estimated when the gate voltage is larger than 0.2 V. The efficiency is almost independent of the Pwell voltage until the saturation point, which is around 300 mV, as shown in Figure 4.

**Figure 5:**
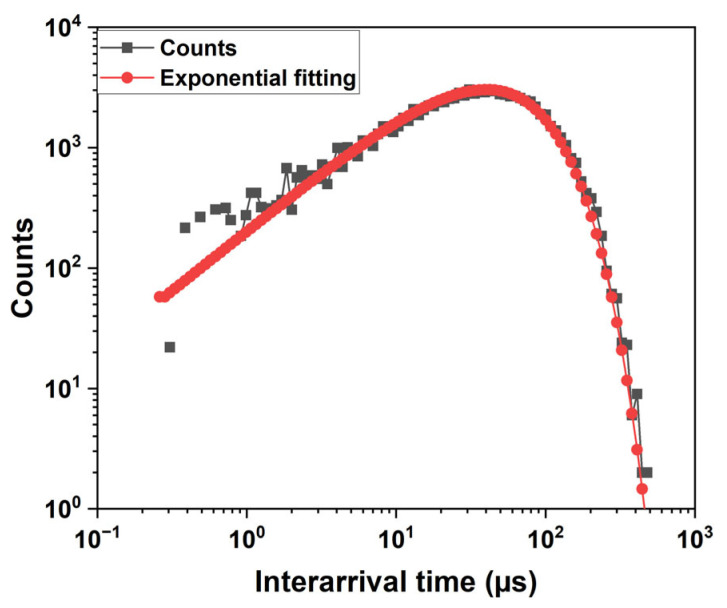
Histogram of the interarrival times (horizontal log scale and log bins), with the exponential fitting of the primary events.

Figure 5 shows the distribution of inter-arrival times between the counts at V_gate_ = 1.8 V and V_Pwell_ = 240 mV. The measurements were performed using a high-speed oscilloscope, acquiring the voltage across a small resistor in series with the quenching resistor. The waveform data were saved from the oscilloscope and processed offline using MATLAB R2023a data analysis software to extract the time intervals between consecutive pulses. This analysis allowed us to construct the histogram shown in the figure. The avalanche pulses generated by electron injection follow a Poisson distribution, which means that the inter-arrival times have an exponential distribution. An exponential function can indeed be used to fit the histogram of inter-arrival times. The exponential models fit very well with the experimental histogram for inter-arrival times larger than 1 μs. At small values of inter-arrival time, an excess of pulses is observed. The difference between the measurement and the fit is due to afterpulsing effects [14].

**Figure 6:**
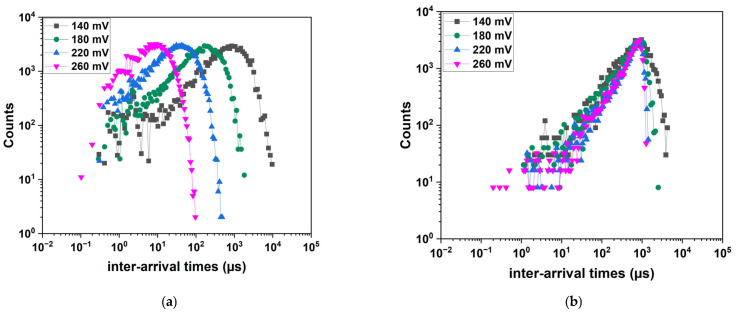
Count rate as a function of the inter-arrival time for different values of the Pwell voltage: (**a**) V_gate_ FET = 1.8 V and (**b**) V_gate_ FE T = 0 V.

The log–log avalanche interarrival time distributions are shown in Figure 6 for V_gate_ = 1.8 V and V_gate_ = 0 V. As the Pwell voltage increases, the count distributions shift towards shorter inter-arrival times, indicating a higher electron flux, as shown in Figure 6a. In contrast, when the FET gate is biased at 0 V, as shown in Figure 6b, the distributions appear distorted and cannot be fitted with exponential characteristics. Since this behavior could not be explained through measurement analysis alone, we conducted device simulations to gain further insights into the underlying mechanisms.

## 3. TCAD Simulations

TCAD simulations were conducted using a drift-diffusion simulator with models tuned for silicon parameters. The models used in the analysis include effective intrinsic density, doping-dependent Shockley–Read–Hall generation/recombination and mobility, and high field saturation, with default values for most semiconductor parameters. Electron and hole lifetimes representative of a typical CMOS imaging process were used. Impact ionization effects are incorporated using the Van Overstraeten and De Man model. A TCAD model of the SEBAT was developed based on the device layout and doping profiles provided by the foundry. To simplify the simulation, a 2D domain was used, and a cylindrical symmetry configuration was employed to estimate the characteristics of a 3D device with a round-shaped layout. In the simulation domain, the diameter of the avalanche region is 7.4 μm, while the diameter of the injector is 0.87 μm.

**Figure 7:**
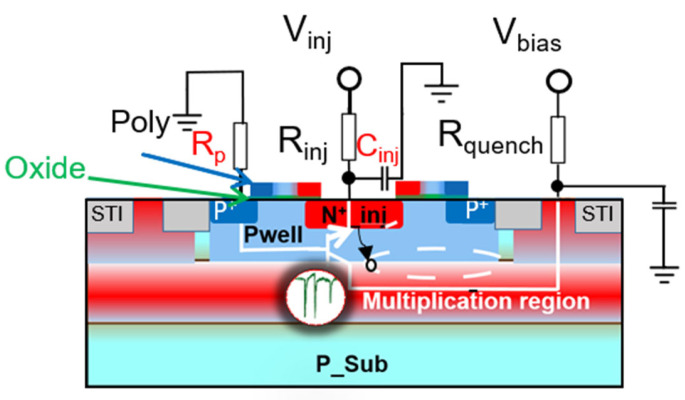
Cross-section of the implemented SEBAT in 150 nm CMOS combined with the external lumped elements.

The model was first validated by comparing the experimental and the simulated Gummel plots. Then, the modeling efforts were directed toward understanding the effect of the functional and parasitic circuit elements on SEBAT operation. A mixed-mode transient simulation was set up, incorporating lumped elements representing parasitic and external components, as shown in Figure 7. The parasitic elements considered in the simulations are base parasitic resistance R_p_, injector capacitance C_inj_, and collector parasitic capacitance C_pc_, which represent the pad capacitance, the wires, and the parasitic capacitance parallel to the quenching resistance. The quenching resistance R_quench_ and the equivalent resistance of the signal source at the injector R_inj_ were also included as a lumped element.

The value of the parasitic base resistance R_p_ was extracted from experimental I-V curves measured on the base-collector junction in forward bias. Since the semiconductor base resistance is estimated around 10 Ω in TCAD simulations, the dominant contribution is due to contact resistance. In fact, the contacts in the device active area, including the base contact, were not silicided to avoid the introduction of generation-recombination centers in the avalanche region, and a small base contact area was drawn. Notably, an accurate estimation of the contact resistance using TCAD is challenging due to complex mechanisms involving hole tunneling, field-induced barrier lowering, and unknown parameters such as the metal work function. The experimental extraction of this parameter ensures a more realistic representation of the device’s electrical characteristics. The extracted value for R_p_ is 357 Ω, which is compatible with the expected resistance for a non-silicide contact. The value of R_p_ = 10 Ω was also considered in simulations to account for a device with optimized base contact resistance. A parasitic collector capacitance C_pc_ of approximately 1 pF and an injector capacitance of 20 fF were estimated through parasitic extraction from the THz-SET layout using the PDK design tools. For the C_pc_ parameter, values ranging from 10 fF to 10 pF were considered as values representative of different integrated and external quenching circuit configurations in the simulation. For R_inj_, a wide range of values was explored to account for the different equivalent resistances of the FET detector when the gate is biased at different voltages and under situations that can be found in different applications. The different values of the lumped elements used in the simulation, including quenching resistance, injector resistance, and several parasitic elements, are reported in Table 1.

Simulations start with a quasi-static analysis, which serves as the initial state for the transient analysis. The output of transient simulations shows the evolution of voltages and currents during an avalanche event, which was triggered by a localized generation in the high-field region.

In the simulation, a negative voltage is applied to the injector, while the Nwell collector was biased at 22 V, a voltage large enough to provide a high triggering probability for electrons. The simulated breakdown voltage (19.2 V) shows good agreement with the measured value (19.5 V).

## 4. SPICE Model

**Figure 8:**
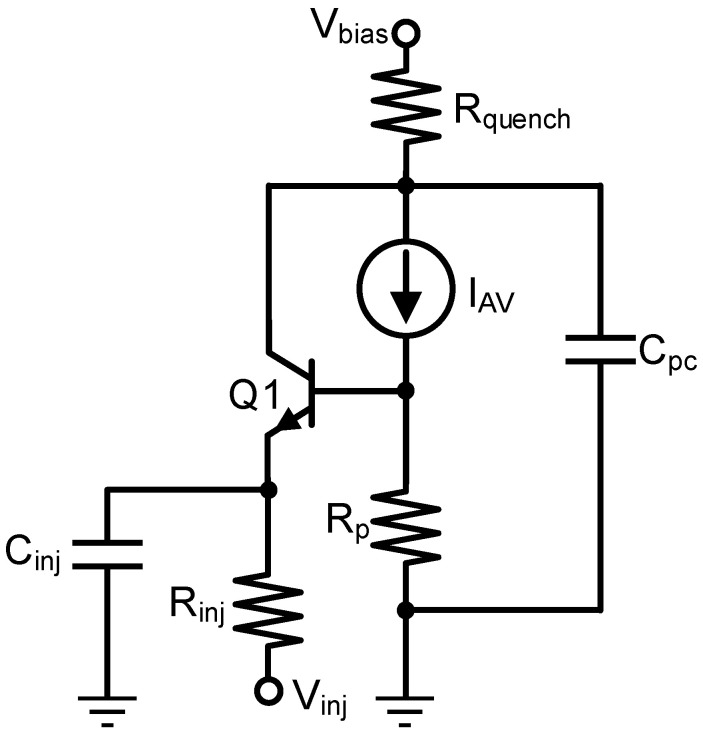
SPICE model of the SEBAT.

As a design tool for the simulation of SEBAT-based circuits, a SPICE model with parameters extracted from TCAD simulations was developed. The SEBAT model, shown in Figure 8, combines a bipolar transistor and a Geiger-mode APD model. The bipolar transistor is modeled after the widely used Gummel–Poon macro model [15]. The Geiger-mode avalanche diode can be modeled in two ways: either with a series branch with a switch, a voltage source, and a resistance component [16] or as a current source [17]. In this work, we opted for the second approach, but the first one could also be adopted and would provide comparable results. The current generator I_AV_ is used to model avalanche current generation in Geiger-mode operation. The SPICE circuit also incorporates the various parasitic and functional components shown in Table 1.

**Figure 9:**
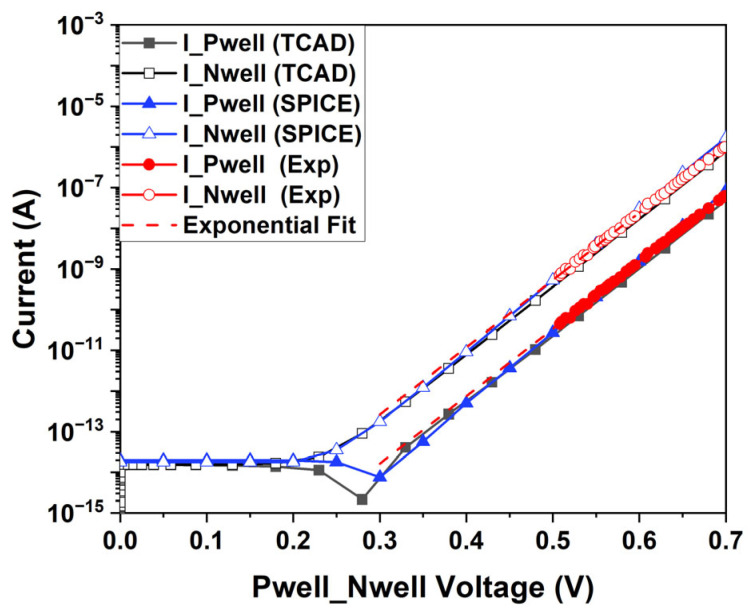
Gummel plot of the SEBAT operated as a conventional bipolar transistor. Experimentally extracted characteristics are compared with the ones simulated with TCAD and SPICE.

Figure 9 shows the Gummel plot of the SEBAT, i.e., the base and collector current measured as a function of the base-emitter voltage, where the emitter was grounded through the MOSFET controlled by Vgate, and a positive voltage sweep was applied to the base (Vpwell). This plot is widely used in the characterization of bipolar transistors to represent the device operation in DC conditions and was measured on a stand-alone test structure fabricated using the same 150 nm CMOS process and the same geometry as the SEBAT used in the THz-FET device. The TCAD model achieves a significant level of agreement with the experimental curves. From this plot, a common-emitter current gain β of approximately 10 was extracted, providing a common-base current gain α of the order of 0.9 [13]. The parameters of the SPICE model were extracted from TCAD simulations, and its corresponding Gummel plot is shown in Figure 9. The analysis was extended to show how the regression fit from the measured data compared against the simulation curve in the region down to approximately 0.35 V, where the exponential model remains valid for these PN junctions (the ideality factor is almost 1). Below 0.5 V, reliable direct measurements of the current could not be performed due to chip packaging and limitations in the measurement setup, where the device could not be properly shielded from environmental noise.

The junction capacitances as a function of voltage were estimated from the TCAD simulation and were included in the model, since they are relevant for transient-mode operation.

## 5. Simulation Results and Discussion

Device transient operation was first analyzed in TCAD and then using the SPICE model tuned according to TCAD data. The analysis was aimed both at the interpretation of experimental data and at the validation of the compact SPICE model. Since the count rate as a function of the base-emitter voltage, as presented in Section 2, shows a different trend when the injector is connected to a high-resistance (FET OFF) or medium-low resistance (FET ON) source (see Figure 6), two sets of simulations for R_inj_ = 2.7 kΩ and for R_inj_ = 10 GΩ are discussed as representative of the two cases. The quenching resistance is set to 10 MΩ, and as long as it remains within this high-resistance range, it does not affect the shape or timing of the avalanche current and injector current. The recharge time of the injector is governed by its own RC time constant (R_inj_ and C_inj_) and is independent on the SPAD recharge time. While SPAD recharge is dominated by the time constant R_quench_·C_pc_, the injector can recharge much faster if the value of R_inj_ is low, such as when using a transistor biased at or above the threshold. Thus, the injector and SPAD recharge times are distinct and independently controlled.

**Figure 10:**
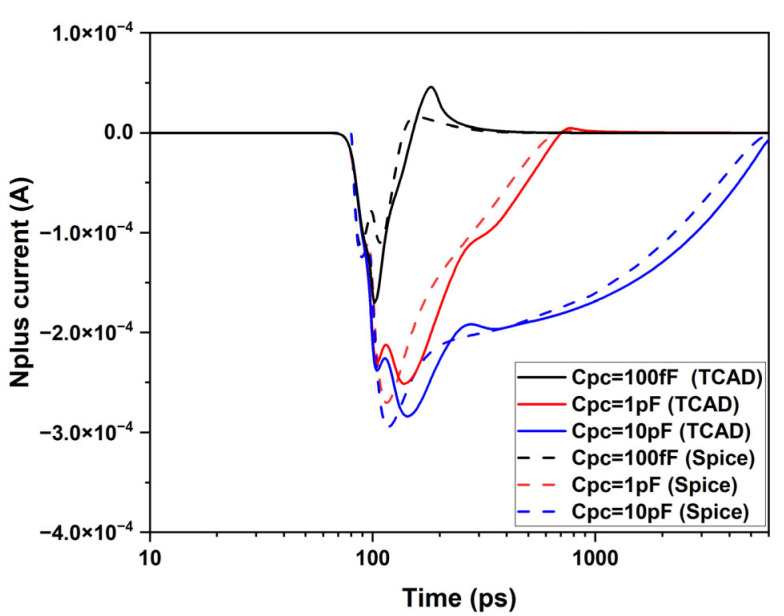
Injector current as a function of time: (a) TCAD simulation and (b) SPICE simulation.

Figure 10 depicts the injector current as a function of time obtained from the TCAD and SPICE simulations. The plot shows the effect of C_pc_ on the duration and magnitude of the injector current pulses with R_p_ = 357 Ω. As C_pc_ increases from 100 fF to 1 pF and then to 10 pF, the pulse duration extends significantly, and a secondary peak appears. The SPICE model captures the same behavior observed in the TCAD simulations, with good quantitative agreement.

The current pulses at the injector observed in Figure 10 stem from the increase in the forward bias of the base-emitter junction, which is caused by the avalanche current flowing into the parasitic resistance R_p_ during avalanche discharge. A larger C_pc_ leads to a larger avalanche charge generated per event and a slower discharge process, which prolongs the duration of the avalanche current pulse. Consequently, the charge injected into the emitter is influenced by both R_p_ and C_pc_. Minimizing these contributions would significantly reduce or even eliminate this charge injection.

**Figure 11:**
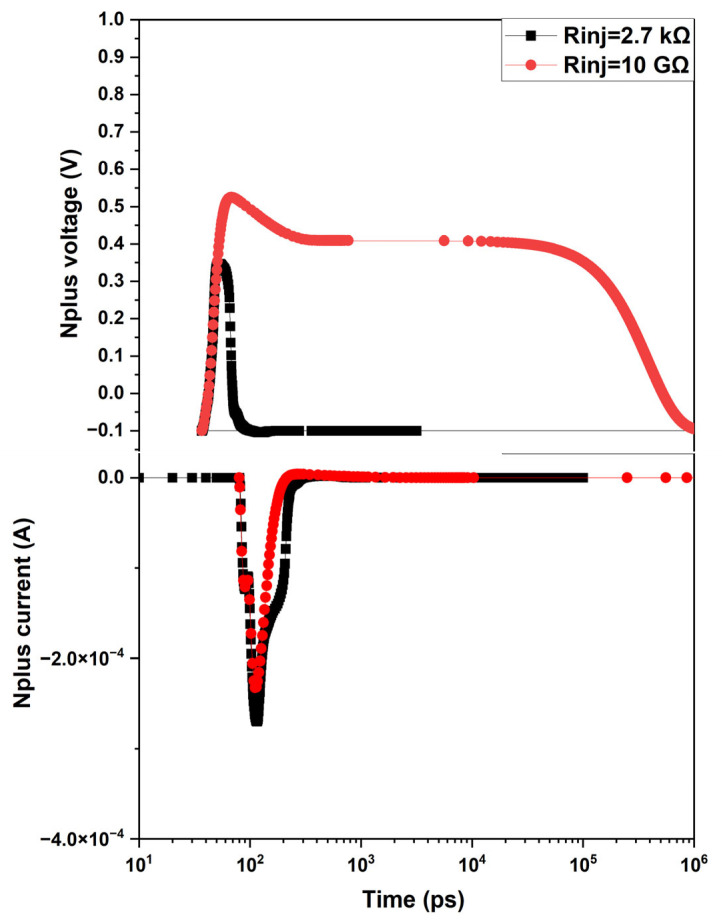
Temporal evolution of injector voltage and current with high and low impedance injector loads.

The effect of R_inj_ on SEBAT transient operation was reproduced by using the SPICE model, as shown in Figure 11, where V_inj_ and the injector current are shown for both low and high values of R_inj_. In both cases, a net charge packet is injected from the emitter region into the base towards the avalanche junction. In the case of medium and low resistance on the injector, this effect does not affect the count rate vs. voltage curves, as shown in Figure 3 and Figure 4 for V_gate_ ≥ 0.2 V, since the duration of the current pulse is of the order of 1 ns and thus shorter than the avalanche junction recharge time. The presence of a transient current at the injector becomes relevant, except when a source with very high resistance is connected to the injector. In this case, the transient current discharges the base-emitter capacitance, and the emitter recharge time is determined by the time constant C_inj_R_inj_. If this time constant is larger than the device recharge time, the base-emitter voltage increases slowly after an avalanche event until it once again reaches the initial value after a few time constants. During the injector recharge time, the count rate will be reduced, and the electron emission statistics will be affected. Therefore, with these considerations, both effects observed experimentally in Section 2 for an applied voltage of V_gate_ = 0, i.e., the reduction in the count rate and the distortion of the inter-event time distribution statistics, can be explained.

**Figure 12:**
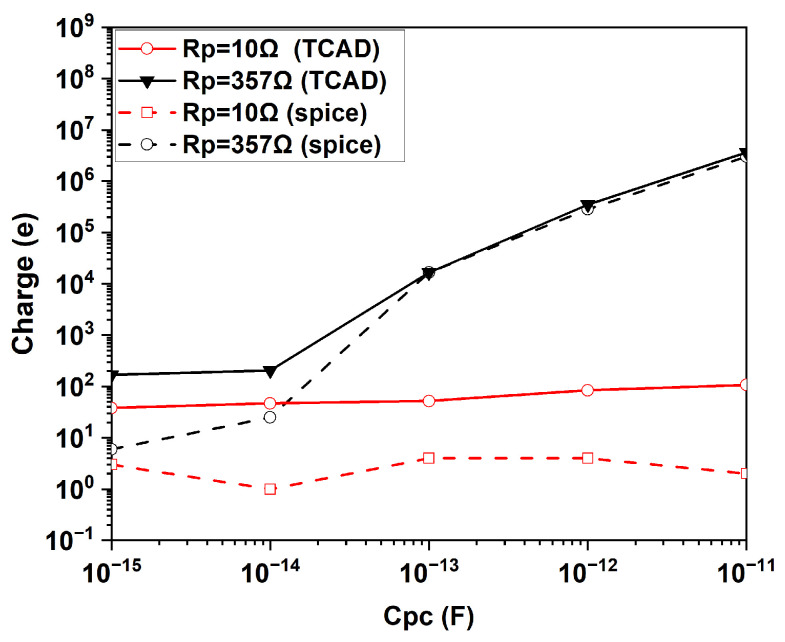
Injected charge per pulse as a function of the capacitance at the collector using TCAD and SPICE simulations.

The effect of collector parasitic capacitance C_pc_ and base parasitic resistance R_p_ on the integrated charge per pulse is summarized in Figure 12**.** As can be observed, by increasing C_pc_ from 10 fF to 10 pF, the number of injected electrons per pulse increases from less than 100 to the order of 10^5^ when R_p_ = 357 Ω. This increase is not observed for a low value of R_p_ = 10 Ω. It is worth noting that, in the case of a very low charge, the number of electrons estimated in the TCAD simulation could be affected by the accuracy of the numerical analysis in computing the integral of the flowing current. Thus, the exact value of the charge flowing at low values of C_pc_ and R_p_ should be considered as a worst-case indication. Therefore, the important message provided by this simulation is that large values of R_p_ and C_pc_ could result in a large instantaneous charge flow at the injector and thus produce a large inefficiency if the SEBAT is used for single-electron detection. This observation aligns with the experimental findings in [11,12].

Figure 12 shows the injected charge per pulse as a function of C_pc_ predicted by SPICE simulations. From this plot, SPICE simulations closely mirror the behavior observed in the TCAD simulations, validating the compact modeling approach also under this point of view. Both simulations exhibit similar trends, where increasing the parasitic capacitance (C_pc_) and base resistance (R_p_) results in higher amplitude and longer-duration injector current pulses. However, an offset can be observed for low values of the injected charge. In the TCAD simulations, the minimum injected charges remain in the range of tens of electrons. In contrast, the SPICE simulations show a reduction in injected charges to values of the order of a single elementary charge.

This discrepancy is primarily attributed to the accuracy of the TCAD simulations, since the integration of the current versus time curves can lead to significant errors in the evaluation of the total flowing charge.

Nonetheless, apart from this discrepancy, the SPICE and TCAD simulations demonstrate excellent agreement, capturing the essential transient behavior of the SEBAT device under varying circuit parameters.

## 6. Conclusions

In this work, the transient operation of a SEBAT was studied using TCAD simulations, and a SPICE compact model was developed for this device. This modeling study explains the behavior of the SEBAT observed in a test device designed for THz radiation detection and offers an interpretation of the low efficiency in electron-to-avalanche pulse conversion reported in some experimental works. This study identifies two key parameters that need to be optimized for SEBAT efficient operation: reducing the base parasitic resistance and minimizing collector parasitic capacitance. The first parameter can be improved by increasing the overall base contact area, while the second one requires SEBAT integration with a low-capacitance quenching element. The optimization of these parameters would significantly enhance the electron-to-pulse conversion efficiency, increasing the SNR of the digitized signal proportionally to the square root of the total counts, since the SEBAT count rate follows a Poisson distribution. In THz detection applications, this enhancement will directly improve the SNR in signal digitization.

The SPICE macro-model presented in this work, which was tuned using TCAD simulations, was able to accurately reproduce the device’s behavior. The consistency between the TCAD and SPICE simulations reinforces the validity of the modeling approach and provides confidence in the ability to predict the transient behavior of the SEBAT device for different values of the parasitic parameters. The proposed SPICE model can thus be employed in the design of optimized SEBAT-based circuits, paving the way for high-sensitivity THzSET detectors.

## Figures and Tables

**Table 1:** Numerical values of the external lumped elements used in mixed-mode TCAD simulations.

Symbol	Quantity	Value
R_p_	Parasitic resistance at the Pwell	357 Ω/10 Ω
R_inj_	External resistance at the injector	1 kΩ–100 GΩ
C_inj_	Parasitic capacitance at the injector	20 fF
R_quench_	Quenching resistance	10 MΩ
C_pc_	Parasitic capacitance at the collector of the SEBAT	10 fF–10 pF

## Data Availability

Data are available upon request.
